# Identifying Potential Ageing-Modulating Drugs *In Silico*

**DOI:** 10.1016/j.tem.2018.11.005

**Published:** 2019-02

**Authors:** Handan Melike Dönertaş, Matías Fuentealba, Linda Partridge, Janet M. Thornton

**Affiliations:** 1 European Molecular Biology Laboratory, European Bioinformatics Institute, Wellcome Genome Campus, Hinxton CB10 1SD, UK; 2 Institute of Healthy Aging, Department of Genetics, Evolution and Environment, University College London, London, UK; 3 Max Planck Institute for Biology of Aging, Cologne, Germany; 4 These authors contributed equally to this work

**Keywords:** ageing, drug repurposing, longevity, computational biology

## Abstract

Increasing human life expectancy has posed increasing challenges for healthcare systems. As people age, they become more susceptible to chronic diseases, with an increasing burden of multimorbidity, and the associated polypharmacy. Accumulating evidence from work with laboratory animals has shown that ageing is a malleable process that can be ameliorated by genetic and environmental interventions. Drugs that modulate the ageing process may delay or even prevent the incidence of multiple diseases of ageing. To identify novel, anti-ageing drugs, several studies have developed computational drug-repurposing strategies. We review published studies showing the potential of current drugs to modulate ageing. Future studies should integrate current knowledge with multi-omics, health records, and drug safety data to predict drugs that can improve health in late life.

## Highlights

The surge of high-throughput technologies over the last decade has promoted the use of computational biology as a method to integrate and interpret the multi-omics information of ageing, diseases, and drugs.

Ageing-related pathways in animal models are also involved in human diseases. Thus, drugs employed to treat human diseases potentially could be repurposed to ameliorate aspects of the ageing process and hence prevent more than one age-related condition in humans.

Recently, several approaches using different data sources have been developed to prioritise prolongevity compounds for humans and for testing in animal models.

The development of biorepositories, longitudinal cohort studies, and electronic health records is strengthening the bridge between the clinical and molecular data, thus enabling future research on human ageing.

## Ageing, Diseases, and Healthspan

Human **life expectancy** (see Glossary) has steadily increased since the middle of the 19th century in many parts of the world [1] and is projected to continue to do so [2]. Longer lives are a result of improved living conditions and medical care, which have made people healthier at most ages 3, 4. However, the years of healthy life (healthspan) have not kept up with the overall increase in life expectancy, and there is a large and growing period of poor function and ill health at the end of life [5]. Ageing is the major risk factor for illness, including the major chronic and killer diseases: cancer, metabolic and cardiovascular disease, and dementia 6, 7. The increasing proportion of unhealthy old people, often with two or more diseases (multimorbidity) 8, 9, 10, and associated problems from the use of multiple drugs for their treatment (polypharmacy) 11, 12, 13, 14, is posing an increasing burden on elderly people, their carers and social networks, and healthcare systems. Reducing the impact of ill health at the end of life is, therefore, a high priority for national governments and international health organisations [15].

## The Malleability of Ageing

Since ageing is the major risk factor for poor functioning and disease, intervening to ameliorate its effects could also prevent multiple age-related conditions simultaneously. There is growing evidence for the feasibility of this approach. People who die when they are very old (100, 105, 110) show progressively less multimorbidity at the end of their lives 16, 17. Thus, a healthy ageing phenotype in humans can be achieved, and if we could understand the mechanisms leading to it, we might be able to extend it to the general population. Additionally, work over the past ∼20 years has shown that environmental, genetic, and pharmacological interventions in animals can extend both their **lifespan** and their healthspan 18, 19. Dietary restriction (DR), a reduction in food intake that avoids malnutrition, can extend lifespan and induce a marked improvement in health during ageing in diverse organisms 20, 21 including rodents. Two studies of rhesus monkeys subjected to DR found that the animals had lowered plasma triglycerides, diabetes, cardiovascular disease, sarcopenia, incidence of neoplasms, and brain atrophy, all features of ageing in humans 22, 23, 24. However, compliance with DR regimes in humans is low, and for this reason, it is not a practical public health intervention.

Changes in diet are monitored by many nutrient-sensing systems, including the insulin/insulin-like growth factor and target of rapamycin (mTOR) signalling network. This highly conserved network senses nutrients, growth factors, and stress status, and modulates the costly activities of the organism, such as metabolism, growth, and reproduction, accordingly. Genetic interventions that reduce the activity of the network have proved to extend lifespan in nematode worms, fruit flies, and mice 18, 19, 25. These long-lived mutants are protected against many natural pathologies of old age and also those associated with genetic models of **age-related diseases**. Genetic variants in **orthologous genes** in humans are associated with survival to advanced ages 26, 27, 28. Mechanisms of ageing are highly conserved during evolution, and the process shows a set of characteristic **hallmarks of ageing** [29], which are also present in the aetiology of age-related diseases 7, 29. Interventions that improve health during ageing and increase lifespan in laboratory animals do so by reducing the impact of one or more of these hallmarks.

Increasingly, attention is turning to the possibility of pharmacological intervention into the ageing process, with a view to preventing age-related diseases. Although, ageing is becoming popular amongst the conditions under study in clinical trials, which aim, for example, to find drugs that restore gene expression levels to that of young healthy subjects (ClinicalTrials.gov identifier: NCT02432287) or reduce ageing-related biochemical parameters (NCT03451006), *de novo* drug development for ageing poses major challenges. We lack good **biomarkers of ageing** that could give a rapid prediction of the outcome of drug treatment, and a clinical trial with a potentially long-term treatment of an initially healthy population would be both prohibitively costly and would require drugs that are almost completely safe and free of side-effects. However, unsurprisingly, many of the gene products identified as potential targets to reduce the impact of ageing are already the targets of drugs licensed to treat specific age-related conditions. For instance, the licensed drug sirolimus (rapamycin), an inhibitor of the mTOR Complex 1 (TORC1), is used to prevent rejection of tissues after transplant, restenosis after cardiac surgery, and to treat cancer. This drug also extends lifespan in yeast, nematode worms, flies, and mice, and prevents many age-related conditions in ageing mice 30, 31, 32. Inhibitors of mTOR can also potentiate the weak response to immunisation against influenza in ageing mice and humans 33, 34. Metformin, a drug used to treat type 2 diabetes, also targets the nutrient-sensing network. This drug has been shown to reduce all-cause mortality in diabetic patients when compared with those receiving nonmetformin therapies, and even non-diabetics in multiple studies [35], and is under trial for protection against the effects of ageing 36, 37.

Repurposing of drugs, to protect against the effects of ageing and hence delay or prevent age-related diseases, is thus an increasingly realistic prospect. Indeed, some existing drugs could already be viewed as being used in this way. Both statins and drugs that lower blood pressure are widely used to prevent cardiovascular disease 38, 39, for which age is the major risk factor. Non-compliance with preventive drug regimens will always limit their efficacy, but there is at least the prospect that many age-related conditions could be warded off pharmacologically by those who wish to do so. There is, therefore, growing interest in identifying and prioritising such potentially **geroprotective drugs**. In this short review, we discuss the available data and some of the bioinformatics methods that are being used to this end. It is important to stress that these are methods to prioritise drugs for experimental testing. They provide the first step of many required for successful **drug repurposing**.

## Using Computational Biology to Discover Anti-ageing Drugs

**Figure 1:**
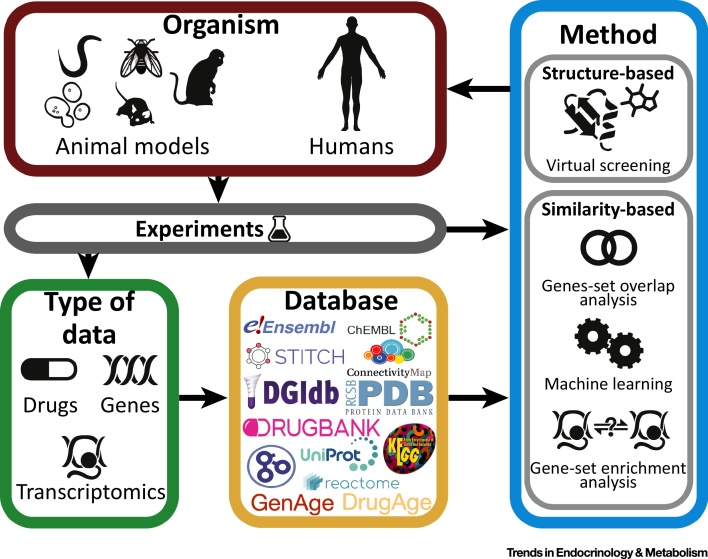
Overview of the Information and Methods Used in the Studies. Databases storing different types of data from experiments in various organisms (source organism) are used to apply drug-repurposing methods to identify ageing-modulators for different organisms (target organism). In some studies, the information from previous experiments is used together with the databases or directly as input for the methods.

Here we review 12 recent publications, summarised in Table 1 and Figure 1, aiming to identify and prioritise **prolongevity drugs** for animal models and humans. All such studies have been enabled by the development of powerful databases for the annotation and curation of genes/proteins (Ensembl [40], UniProt [41]), their associated functions and pathways (Gene Ontology [42], KEGG [43], Reactome [44]) and chemical ligands and drugs interacting with them (ChEMBL [45], DrugBank [46], STITCH [47], drug gene interaction database; DGIdb [48], protein data bank; PDB [49]) or affecting their expression (Connectivity Map [50], CREEDS [51]), as well as drugs (DrugAge [52], Geroprotectors.org [53]) and targets (GenAge [54], Aging Clusters [55]) implicated in ageing and age-related disease mechanisms (Figure 1).

**Table 1:** Published Studies of Drug-Repurposing to Target Ageing

Study	Source organism^a^	Source of data^b^	Method^c^	Target organism^d^	Additional data^e^	Refs
Virtual screening against known ageing genes						
1. Snell (2016)	Rotifer	Specific genes^f^	Virtual screening	Rotifer	* Drugs (FDA approved in DrugBank & ZINC8 [76]) * Protein structure (PDB)	[56]
2. Snell (2018)	Rotifer orthologues of yeast, worms, flies, mice	GenAge	Virtual screening	Rotifer	* Drugs (DrugBank, ZINC8 [76]) * Protein structure (PDB)	[57]
3. Mofidifar (2018)	–	Specific genes^g^	Virtual screening and molecular dynamics	Human	* Drugs (FDA approved in DrugBank) * Protein structure (PDB)	[59]
Similarity-based approaches						
Finding drugs that target known ageing-related genes						
4. Fernandes (2016)	Human orthologues of model organism genes	GenAge	Gene-set overlap analysis	Human	* Protein–drug interaction network (DGIdb) * Orthologues (Ensembl Compara)	[60]
5. Fuentealba (2018)	Human	Ageing Clusters	Gene-set overlap analysis	Human	* Protein–drug interaction network (STITCH) * Protein–protein interactions (STRING [77]) * Functional annotations (GO) * Pathways (KEGG & Reactome)	[61]
Finding drugs similar to known prolongevity drugs						
6. Liu (2016)	Worm	High-throughput drug screening in *C. elegans*[63] and GenAge	Machine learning	Worm	* Protein–drug interaction network (*STITCH*)	[62]
7. Barardo (2017)	Worm	*DrugAge* and High-throughput drug screening in *C. elegans*[63]	Machine learning	Worm	* Drugs (DGIdb) * Functional annotations (GO) * Chemical descriptors (MOE)	[64]
Comparing transcriptome signatures of ageing and drugs						
8. Calvert (2016)	Human orthologues for rat and macaque genes	Caloric restriction expression signature [78]	Gene-set enrichment analysis	Human	* Drug-induced expression profile (CMap)	[65]
9. Dönertaş (2018)	Human	Age series expression	Gene-set enrichment analysis	Human	* Drug-induced expression profile (CMap)	[66]
10. Yang (2018)	Human	Young and old expression	Gene-set enrichment analysis	Human	* Drug-induced expression profile (CREEDS)	[67]
Approaches to prioritise drugs for testing						
11. Aliper (2016)	Human	Young and old expression	Pathway similarity	Human	* Anti-ageing drugs and their targets (http://www.geroprotectors.org) * Ageing-related pathways	[68]
12. Ziehm (2017)	Human	GenAge and GO ageing term (GO:0007568) [42]	Empirical scoring function	Worm and fly	* Protein structure (PDB) * Sequence (Uniprot) * Binding affinity (RF-Score [79]) * Bioavailability assay [80] * Purchasability (ZINC [76], eMolecules; https://www.emolecules.com) * Drug approval (ChEMBL, DrugBank) * Orthologues (Ensembl Compara)	[69]

GO, Gene ontology; MOE, Molecular Operating Environment [http://www.chemcomp.com/MOE-Molecular_Operating_Environment.htm].

a Organism from which the ageing information was acquired.

b Source of ageing knowledge (e.g., ageing databases or age-related expression data).

c See Box 1 for the detailed information.

d Organism in which the drugs identified in the study should have an effect.

e Additional information used in the method.

f TRP7, S6K, FhBC.

g AMPK.

Although the published studies of drug repurposing to target ageing use different strategies and sources of data, they can be classified into two main categories: methods employing the structural information to predict drugs potentially interacting with proteins already identified as being involved in ageing, and methods based on the similarity between ageing-related drugs or genes based on molecular structure, interactions, pathways, or networks (Table 1).

## Virtual Screening against Known Ageing Genes

The concept here is to find drugs which are known to target those genes that have been implicated in ageing. Two studies adopted methods based on the hypothesis that proteins or ligands with similar structures are likely to bind similar ligands or proteins, respectively, to predict drug-target interactions. The first of these (Study #1) [56] aimed to identify novel drugs targeting three specific temperature-sensing proteins implicated in ageing in the rotifer *Brachionus manjavacas* (TRP7, S6P, FhBC). The authors used a virtual screening (Box 1) software called FINDSITE^comb^ that combines protein modelling with sophisticated threading approaches to model the target. The pockets in the model are then compared with the pockets in experimentally determined structures of proteins with ligands or modelled structures with known binders. The ligands of the top 100 ranked pockets are then compared with a library of screened ligands and ranked by ligand similarity. The authors screened 1347 FDA approved drugs *in silico* and tested four drugs for each target experimentally in the rotifers for their effects on lifespan and healthspan. Out of the 12 compounds tested, 5 significantly increased the rotifers’ lifespan. Changes in healthspan, approximated by swimming speed, reproduction, and mitochondrial activity, were also observed. In a subsequent study by the same authors (Study #2) [57], the number of proteins analysed was expanded to include a set of ageing-related genes found in other animal models that are orthologous to genes in rotifers. This time a total of 94 targets were screened *in silico* using the FINDSITE^comb^ software. The top 1% binding compounds for each target were further ranked by their cumulative lifespan extension achieved by genetic interventions into their targets as taken from experimental model organism data, and filtered according to availability and previously predicted side effects [58]. From the 31 drugs experimentally tested in rotifers by two 10-day survival screens, seven drugs were further tested in two whole-life survival analyses, two of which resulted in a median lifespan extension of 13–42%. The prolongevity effect of these drugs was observed even when drug treatment was initiated in middle age.

Box 1 **Structure- and Similarity-Based Methods**

Virtual screening: computational approaches used to identify which molecules among a large collection are most likely to bind to a desired protein of interest (target). There are three categories of screening techniques: (i) ligand-based methods, ranking candidates by their similarity to known ligands of the target; (ii) structure-based methods, docking candidate molecules into the binding site of the target to calculate their binding affinity; and (iii) hybrid methods, using both structure and ligand similarity (e.g., FINDSITE^comb^). These methods use the similarity between the binding pockets of the target and other proteins to identify potential binding molecules. The similarity with these molecules can be then used to rank a desired library of candidate molecules. (Studies #1–3 56, 57, 59).

Molecular dynamics: computational method that uses the equations of motion to simulate the interactions between atoms within a system over time. These techniques are dependent on a ‘force field’, which is a mathematical description of how the atoms/molecules will interact. In biology, dynamic simulations of complexes can be used to assess the changes in binding energy over time and the contribution of specific amino acids to binding. (Study #3 [59]).

Gene-set overlap analysis: using a set of genes associated with a trait of interest (e.g., ageing), the analysis determines which molecules target more of these genes than expected by chance. Similar analyses can be performed using functional annotations and pathways instead of genes. (Studies #4,5 60, 61).

Machine learning to identify candidate drugs: method to predict new drugs based on a set of features in the training set. Training set comprises drugs or targets, fully (supervised) or partially (semisupervised) labelled for their ageing relevance. Starting from a positive set of those drugs that increase lifespan and a negative set of those that do not affect lifespan, a machine learning algorithm learns the features (i.e., those characteristics that most effectively produce a correct classification, such as protein interactions, chemical descriptors, or functional annotations). Based on these features, the algorithm can predict the effect of any new drug on lifespan. (Studies #6,7 62, 64).

Gene-set enrichment analysis: using an expression signature describing the gene expression changes associated with a trait (e.g., ageing), and gene expression profiles induced by different molecules, the analysis calculates a Kolmogorov-Smirnov based test statistic to rank drugs that reverse or mimic the trait signature. Statistical significance is calculated by random permutations of the gene lists. (Studies #8–10 65, 66, 67).

Alt-text: Box 1

Another *in silico* screening study (Study #3) [59] was restricted to a single gene, AMP-activated protein kinase (AMPK), activation of which partially mediates the effects of DR on ageing. To find new molecules to activate AMPK and theoretically mimic DR, Mofidifar *et al*. [59] performed virtual screening (Box 1) using molecular docking of 1908 FDA approved drugs. The interaction between the top-ranked compounds and their targets was then further checked by more detailed molecular dynamics (Box 1). The study reported four compounds with predicted high affinity for AMPK, but these were not tested experimentally.

## Similarity-Based Approaches

Using *a priori* information on known ageing-related genes, prolongevity drugs, or **gene expression profiles**, several studies have implemented a series of similarity-based approaches to identify novel anti-ageing drugs.

### Finding Drugs That Target Known Ageing-Related Genes

Given that drugs targeting ageing-related gene products are expected to affect the ageing process, Fernandes *et al*. (Study #4) [60] focused on finding drugs that target human genes which have orthologues associated with longevity in animal models. The drugs were ranked by the likelihood of targeting ageing-related genes among all targets. For this calculation, only inhibitory drugs interacting with **antilongevity genes** and activators targeting **prolongevity genes** were considered. In total, 376 drugs were obtained, of which, 20 were considered to be statistically significant. Thirteen targeted histone deacetylases, and three were previously associated with lifespan extensions in animal models. Recently, Fuentealba *et al*. (Study #5) [61] used a composite set of ageing-related genes with direct evidence for influencing human ageing, together with physical and functional drug–protein interactions, to implement a similar gene-set overlap analysis (Box 1). Study #5 also considered other levels of biological actions, including pathways, functions, and protein–protein interactions. Three of the top 10 compounds that ranked highest on an aggregate score were previously shown to increase lifespan in animal models, and seven had been proposed to affect longevity by other drug-repurposing methods. The prolongevity effects of the top-ranked compound (tanespimycin) was experimentally validated in *Caenorhabditis elegans*.

### Finding Drugs Similar to Known Prolongevity Drugs

An alternative approach is to find drugs similar to known prolongevity drugs using machine learning (Box 1), which is a strategy well-suited for prediction tasks. Liu *et al*. (Study #6) [62] attempted to predict new prolongevity drugs for *C. elegans*. They adopted a semisupervised algorithm trained with high-confidence prolongevity drugs derived from an experimental screen for *C. elegans* [63], together with their associated ageing-related genes curated from the literature and the GenAge database [54]. They produced a rank-ordered list of 785 drugs with a potential to increase lifespan in worms, with experimental validation for one drug in their list, using a lifespan assay. A separate machine learning approach (Study #7) [64] was trained with chemical descriptors of known prolongevity drugs and functional annotation of their targets. Using a supervised algorithm (i.e., random forest), they generated a ranked list of drugs predicted as lifespan-extending compounds, although no validation was performed.

### Comparing Transcriptome Signatures from Ageing and Drugs

The Connectivity Map Resource provides drug-induced expression profiles for 1309 compounds. Comparing these profiles with ageing-related **gene expression signatures** using a gene-set enrichment analysis (Box 1) can reveal drugs that generate changes in expression correlated (positively or negatively) to those seen in ageing (or any other biological process or disease). This approach requires no *a priori* list of ‘ageing genes’ and can therefore potentially identify new targets, based solely on expression profile similarities. The first study (Study #8) [65] used DR expression profiles in rats and rhesus monkeys to find DR mimetics. They identified 11 drugs that could potentially increase lifespan by mimicking DR. They experimentally tested several of the drugs in *C. elegans* and found that most extended lifespan. Another study (Study #9) [66] used a meta-analysis of gene expression changes in the ageing human brain to identify robust gene expression changes in ageing and find drugs targeting those genes. Using the Connectivity Map data, the authors identified 24 drugs and provided *in silico* validation by showing significant enrichment of known prolongevity drugs in their list. Importantly this data-based approach can identify novel drugs and genes, not previously associated with ageing. Yang *et al*. (Study #10) [67] used a network-based methodology, called ANDRU (ageing network-based drug discovery). Instead of relying on model organisms, this approach was also driven by human transcriptome data (GTEx) from young and old adipose and artery tissues and signatures from the CREEDS database [51] to identify differentially expressed genes within the ageing-related networks and drugs reversing these changes. They report three distinct drugs ranking in the top five. Although none is previously reported as a lifespan modulator, these drugs target pathways that change in expression with age, such as metabolic enzymes and lipid metabolism.

## Approaches to Prioritise Drugs for Testing

One of the major challenges to developing anti-ageing drugs is experimental validation. Since clinical trials involve many ethical considerations and are very expensive, such drugs are pretested in model organisms.

In this spirit, Aliper *et al*. (Study #11) [68], aimed to predict which prolongevity drugs previously tested in *C. elegans* could work in humans. Using young and old human stem cell expression profiles and an algorithm called Geroscope that maps the gene expression changes with age to ageing-related signalling pathways, they ranked a set of candidate drugs by their likelihood of targeting these pathways. To do this they calculated the pathway activation strength for each drug. They shortlisted ten compounds with prolongevity effects in *C. elegans*, and tested six of them for geroprotective effects in senescent human fibroblast cultures. While the majority of tested drugs improved senescence-associated phenotypes, one drug (PD-98059), a highly selective MEK1 inhibitor, also showed life-prolonging and rejuvenating effects.

Comparably, to assess which ‘human’ drugs and chemicals are likely to modulate the *C. elegans* and *Drosophila melanogaster* orthologue of the target, Ziehm *et al*. (Study #12) [69] developed a method to rank chemicals binding to genes implicated in human ageing. They generated an empirical scoring function that considers the conservation of the domain and binding site at the sequence level between the animal and the human protein, and predicted binding energy for the compounds for the human targets and experimental **bioavailability**, in addition to scores for drug-likeness, promiscuity, purchasability, and development status. Although the authors provided no experimental validation, they conducted a comprehensive literature-mining and molecular-docking procedure to validate their results.

## Comparing the Results of These 12 *In Silico* Studies

**Figure 2:**
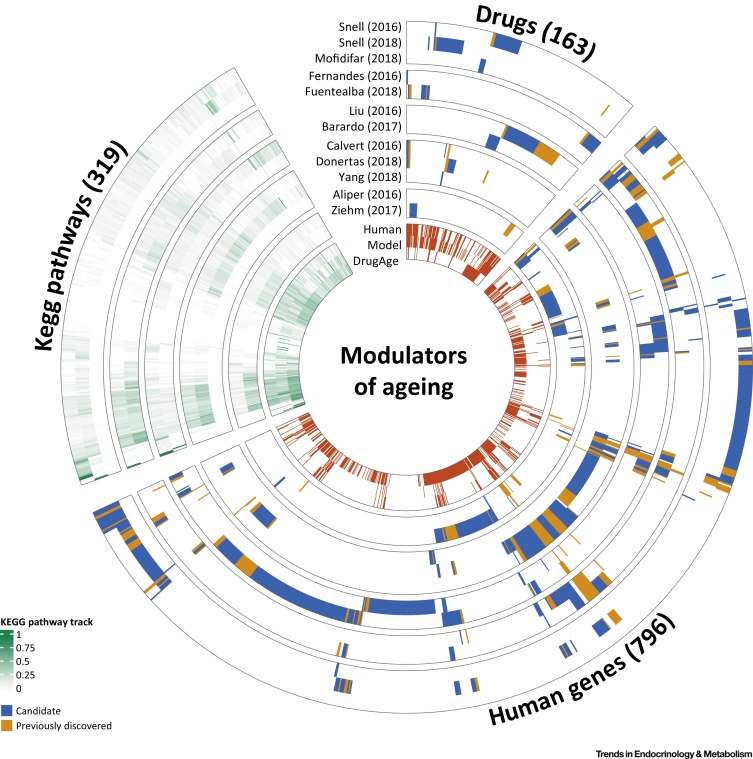
Key Figure: Drugs, Human Genes, and KEGG Pathways Discovered in the 12 Studies

The studies described above had different aims, methods, and data sources. To facilitate their comparison, we have summarised each study in terms of: (i) the drugs identified, (ii) the genes targeted by these drugs, and (iii) all biological pathways (KEGG) known to be targeted by drugs (Figure 2, Key Figure). Additionally, we compared the results with the manually curated databases of ageing-related genes (GenAge) and drugs (DrugAge).

### Drugs

**Figure 3:**
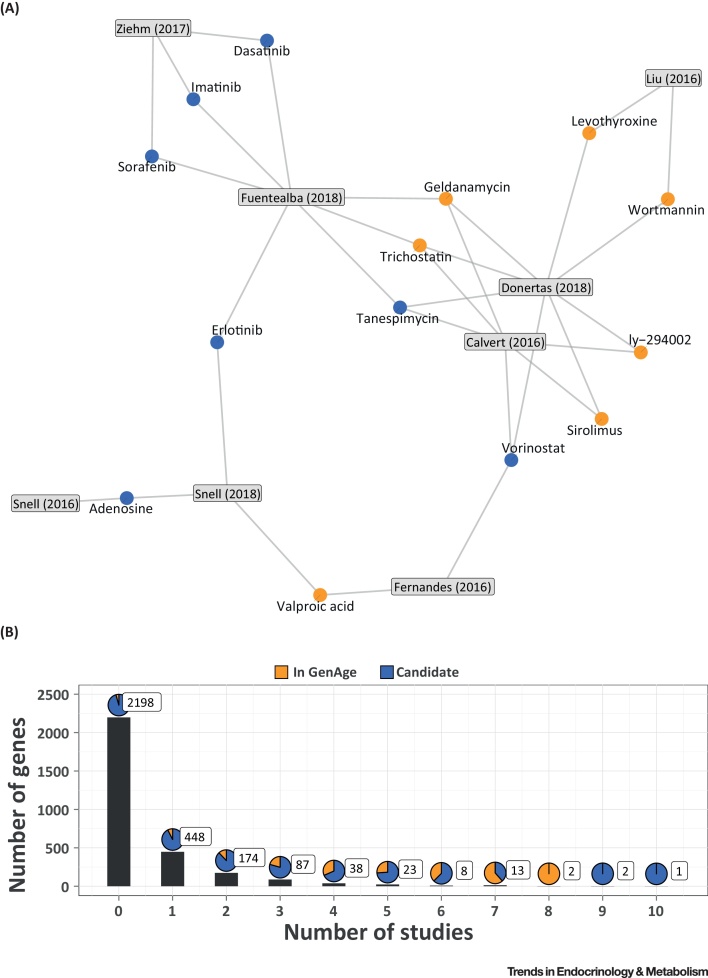
Candidate Drugs and Genes from the Druggable Genome Proposed by Multiple Studies. (A) Network representation of candidate drugs discovered by multiple studies and the studies in which they were found. Orange nodes show drugs previously discovered to affect lifespan in animal models (DrugAge), blue nodes show the novel candidates. The identified drugs are linked to the relevant study. (B) Distribution of the number of genes targeted by the identified drugs with respect to the number of studies. The x- and y-axes show the number of studies and genes, respectively. Some genes in the GenAge are not targeted by any novel candidates (0 studies). The pie charts show the percentage of genes in GenAge (human database) for each category. The boxed numbers show the total number of genes in each category.

Overall only 12% of all DrugAge drugs are prioritised by at least one study (41 of 346 drugs in DrugAge), with one in every four drugs discovered already present in DrugAge, reflecting the prioritisation process and the low number of drugs reported as significant by each study (15 drugs on average). In addition, the 163 drugs identified usually differ between studies, with 91% (149 drugs) of them identified just by one study. From the remaining 14 drugs present in more than one study, trichostatin, geldanamycin, tanespimycin, and vorinostat were identified by three studies (Figure 3A) and, while only the first two are present in the DrugAge database, the remaining two have been experimentally validated for prolongevity effects in animal models 61, 70. Most studies resulted in a list of drugs containing mainly novel candidates not present in DrugAge (122 drugs were classified as novel discovery), the only exception being Aliper *et al*. [68], which focused only on a set of known prolongevity drugs. We also note that 66% of the 122 drugs (i.e., 81 drugs) known to target ageing-related proteins were prioritised by the computational studies reviewed above, as expected considering that these drugs are included in some of the databases used by some of the methods during the prioritisation process.

### Genes

Overall, 34% of the GenAge human genes (103 genes) and 10% of the GenAge model organism genes (94 genes) were identified in at least one study, reflecting at least in part the different sizes of the datasets, with more than three times the number of model organism genes in GenAge. For clarification, the computational methods identified candidate drugs (which are predicted to modulate ageing) amongst the known drugs, most of which are currently used as therapy for a specific disease.

Based on the DGIdb database [64], 27% of the druggable genome (i.e., 796 genes) is targeted by at least one of the drugs identified in the computational studies (Figure 3B) and, while few genes were identified in multiple studies, some of them were present in the GenAge database [54]. Two of these genes, *DDIT3* (DNA Damage Inducible Transcript 3) and *ERBB2* (Erb-B2 Receptor Tyrosine Kinase 2), were targeted by the drugs prioritised in eight studies. However, nine studies also identified drugs targeting *BIRC5* (Baculoviral IAP Repeat Containing 5) and *KRAS* (KRAS Proto-Oncogene, GTPase), and ten studies predicted drugs modulating *ABCB1* (ATP Binding Cassette Subfamily B Member 1), which have not previously been related to human ageing. Despite this, genes discovered by multiple studies do not necessarily suggest higher relevance to ageing, and may instead reflect research bias (e.g., genes targeted by many drugs because of a role in prevalent disease such as cancer). We also observed that 80% of known prolongevity drugs (i.e., 122 of 152 drugs with known targets) target at least one gene that is targeted by the candidate geroprotective drugs identified by these 12 computational studies.

### Pathways

Intriguingly, among the 319 druggable KEGG pathways, 92% include at least one gene that is targeted by the drugs identified in the studies in Table 1. The same tendency was observed for genes in GenAge (83% Model GenAge and 74% Human GenAge), or genes targeted by the DrugAge drugs (88%). While this may suggest ageing is ubiquitous and affects all pathways, another possibility is that genes present in many pathways could be discovered repeatedly because they play a central role in diseases and regulatory mechanisms. Although this may not conclusively prove that ageing is ubiquitous, the prioritised candidate drugs clearly have a genome-wide effect.

## Concluding Remarks and Future Perspectives

Although many caveats need to be considered (Box 2), these 12 computational studies have revealed many currently used drugs with a high potential to modulate lifespan in humans. However, the challenge today is not to extend lifespan (which is happening anyway), but rather to improve late life health by reducing the multimorbidity associated with old age. This challenge has not yet been adequately addressed, either in model organism research or in clinical studies. Many questions remain unanswered (see Outstanding Questions).

Box 2 **Caveats and Caution for In Silico Predictions of Candidate Geroprotective Drugs**

The *in silico* methods are the first step in the process of repurposing drugs for ageing. They prioritise drugs already in the clinic, usually developed against a specific disease, for experimental testing of their effects on ageing. To date there has been only limited experimental validation of the effects of these prioritised candidates, usually limited to one or two chosen examples. The lack of high-throughput validation means that these lists need to be made open access, so that tests can be conducted in many laboratories worldwide. The lack of a complete understanding of the underlying molecular processes involved in ageing, often combined with a lack of knowledge of specific molecular targets for the drugs, complicates the ranking of drugs. The candidate drug predictions are only as good as the data used to derive them. Many of the methods have limited coverage of genes, or include known ageing genes as part of their input, so have some circularity in deriving ageing targets. The expression data analysis is an exception to this, using expression data as a surrogate to compare the effects of ageing and drugs, without other prior knowledge. In future we can expect the equivalent for proteomics or metabolomics data, providing good coverage at several molecular levels. Unfortunately, there is a lack of robust validated data of all types, with only a limited number of longitudinal cohorts with a wide age range and robust molecular measurements. In addition, to date there has been a limited integration of molecular and clinical data, but this is changing. In general, the technological issues (e.g., which machine learning algorithm to use) are not the road block, rather the complexity of the ageing process and associated networks and the confidence in the available data. It is well known that transferring drugs between organisms often gives different outcomes and such effects are rarely predictable at this time. The hallmarks of ageing are often cellular or physiological processes, so integrating with molecular data is challenging. Lastly, designing appropriate clinical trials to test the effects of drugs on ageing and multimorbidity in humans is still in its infancy, considering side effects and appropriate dosage regimes to be explored. The use of previous clinical trials data (designed for different diseases and outcomes) in meta-analyses may provide a valuable *in silico* check before embarking on a large expensive experimental clinical trial.

Alt-text: Box 2

Although some of the potential candidate drugs have been tested for effects on longevity in model organisms, the effects on late life health in humans are difficult to assess experimentally. To do this, access to human health data in old age, combining studies, and allowing in-depth statistical analyses, including consideration of side effects, will be critical. This area is opening up, but a challenge still remaining is gaining access to the clinical data, whilst of course retaining patient confidentiality. The new UK Biobank [71] holds great promise, with data from about half a million individuals, including clinical phenotype data and genotyping, with exome sequencing in progress. These data will be even more powerful when combined with molecular information and data from **longitudinal cohort studies**. An important initiative for testing drugs in model organisms is the National Institute on Aging Interventions Testing Program [72], a multi-institutional program where researchers test the effect of chemical or environmental perturbations on the lifespan of a genetically heterogeneous mouse model. The workflow is designed to increase the reproducibility and find reliable candidates to modulate ageing. However, although we have begun to document the effect of specific pathways on the ageing process and lifespan, we still lack an overall understanding of ageing processes, limiting our ability to use well-established drug discovery methods, such as **genome-wide association studies**, which to date have only provided limited insights [7].

Although conservation of the ageing-related processes and genes amongst organisms is widely accepted, the ability to translate the effects in model organisms to reliable predictions for humans remains a challenge. The need for biomarkers of ageing is becoming ever more important to allow a rapid assessment of the effects of a drug on an organism other than by conducting lengthy, expensive lifespan experiments. The emergence of several **‘epigenetic ageing clocks’** 73, 74, 75 provides opportunities to actively monitor ageing in both cells and organisms. In parallel, high-throughput cellular imaging will allow improved screening and testing.

Clearly, this area of research is expanding rapidly, and the studies described above are just the beginning. Obviously, all this computational work just prioritises candidates before experimental testing, but the methods provide an overview of the current genomic, health, and drug landscape, which can better inform the choice of the best candidates. We can expect a flood of both experimental ageing data on model organisms and clinical datasets for human ageing in the near future. A common workflow will be the integration of many types of data from many studies using computational approaches, which will become more sophisticated and complex. Machine learning approaches, which are increasingly prevalent, will also have an impact here, if sufficiently robust, reliable data can be garnered. Indeed, such analyses will allow us to process relatively noisy, unstructured data more effectively. The hope is that these *in silico* approaches will not only increase our understanding of the processes of ageing but also provide a route towards tackling multimorbidity and improving health in late life.

Outstanding Questions Will drugs that extend lifespan in model organisms translate effectively to improve late life health in humans?

Can we predict the effects of a drug considering its mechanism of action and evolutionary relationships?

What are the best approaches to test such drugs experimentally in humans and avoid serious side effects? There is a need to develop both *in silico* and *in vitro* schemes to facilitate this translation process.

Can we predict the side effects of drugs that will be used in primary prevention by healthy individuals for a long term? Will they be suitable for elderly frail patients?

Will a combination of molecular and clinical data reveal the causes and effects of ageing and allow us to differentiate cause and effect?

Will the most appropriate drugs replicate our responses to ageing or target its causes?

What are the robust biomarkers of ageing and what are the biological mechanisms making them good biomarkers?

Can we utilise epidemiological studies and clinical data to study the effects of drugs on healthspan?

How will we improve access and integration of clinical data across studies and countries?

Molecular data have traditionally been stored in open access data resources available to all. By contrast, personal data is under controlled access depending on patient consent and is often difficult or impossible to access. Can we evolve a robust system to allow optimal use of available data globally, whilst retaining patient confidentiality, to address the medical challenges of an ageing population?

## Glossary

**Age-related diseases**: conditions and illnesses that have an increased incidence with age.
**Antilongevity gene**: genes whose overexpression reduces lifespan; or their reduced expression due to knockout, mutation, or RNA interference, increases lifespan.
**Bioavailability**: the proportion of the intact drug that enters the systemic circulation in the body.
**Biomarker of ageing**: molecular or phenotypic measures that can reliably predict biological age, which might be different from chronological age.
**Drug repurposing**: the use of drugs or compounds that are already in use or known to target other conditions.
**Epigenetic ageing clock**: one of the molecular biomarkers of ageing, which predicts biological age based on DNA methylation levels of a selected CpG site.
**Gene expression profile**: a measure of the RNA products of multiple genes simultaneously.
**Gene expression signature**: robust gene expression patterns that define a condition.
**Geroprotective drug**: (anti-ageing drug) a drug ameliorating the effects of ageing.
**Genome-wide association studies**: a method to associate genome-wide genetic variations with trait-of-interest, through comparison of different individuals.
**Hallmarks of ageing**: the nine characteristics of ageing, suggested by López-Otín *et al*. [29], namely genomic instability, telomere attrition, epigenetic alterations, loss of proteostasis, deregulated nutrient sensing, mitochondrial dysfunction, cellular senescence, stem cell exhaustion, and altered intercellular communication.
**Life expectancy**: the average age an organism is expected to live, based on birth year, age, and other demographics such as country and gender.
**Lifespan**: the length of life of an organism.
**Longitudinal cohort study**: studies where the same type of data is collected for the same subjects over a period of time.
**Orthologous genes**: genes that share a common ancestor and that are present in different species.
**Prolongevity drug**: a drug that extends lifespan.
**Prolongevity gene**: genes whose overexpression extends lifespan; reduced expression reduces lifespan.
